# Adjusted Kolmogorov Complexity of Binary Words with Empirical Entropy Normalization

**DOI:** 10.3390/e28020176

**Published:** 2026-02-03

**Authors:** Brani Vidakovic

**Affiliations:** Statistics Department, Texas A&M University, College Station, TX 77843, USA; brani@tamu.edu; Tel.: +1-979-845-3141

**Keywords:** Kolmogorov complexity, entropy normalization, algorithmic randomness, universal prior, 68Q30, 60G09

## Abstract

Kolmogorov complexity of a finite binary word reflects both algorithmic structure and the empirical distribution of symbols appearing in the word. Words with symbol frequencies far from one half belong to smaller combinatorial classes and therefore appear less complex under the standard definition. In this paper, an entropy-normalized complexity measure is introduced that divides the Kolmogorov complexity of a word by the empirical entropy of its observed distribution of zeros and ones. This adjustment isolates intrinsic descriptive complexity from the purely combinatorial effect of symbol imbalance. For Martin–Löf random sequences under constructive exchangeable measures, the adjusted complexity grows linearly and converges to one. A pathological construction shows that regularity of the underlying measure is essential. The proposed framework connects Kolmogorov complexity, empirical entropy, and randomness in a natural manner and suggests applications in randomness testing and in the analysis of structured binary data.

## 1. Introduction

Kolmogorov complexity, introduced in the seminal works of Kolmogorov [1,2], Zvonkin and Levin [3], and Levin [4], provides an algorithmic notion of complexity for finite objects and infinite sequences. A binary word is regarded as complex if the length of the shortest program that outputs it on a universal machine is essentially equal to the word length itself, whereas highly structured words admit substantially shorter descriptions.

A classical and well-known observation is that Kolmogorov complexity is strongly influenced by the empirical distribution of symbols. For a word of length *n* containing $n_{1}$ ones, the logarithmic size of the corresponding combinatorial class is approximately nH(p), where $p=n_{1}/n$ and *H* denotes binary entropy. As H(p) varies with symbol frequencies, raw Kolmogorov complexity conflates intrinsic algorithmic structure with the purely combinatorial effect of symbol imbalance. Words with extreme imbalance appear less complex, even when no additional structure is present.

This paper addresses this issue by comparing description length to the combinatorial baseline implied by empirical symbol frequencies. The guiding principle is that descriptive structure should be assessed relative to the size of the empirical type class rather than relative to word length alone. At the level of finite words, this baseline is explicit and quantifiable through empirical entropy, making it possible to separate frequency effects from intrinsic algorithmic regularity before any asymptotic limit is taken.

Building on this idea, we introduce an entropy-normalized formulation of Kolmogorov complexity that rescales description length by the empirical entropy of the observed symbol distribution. This normalization was already suggested in Zvonkin and Levin [3], but without a systematic analysis or connection to algorithmic randomness. The present work develops this idea into a coherent framework, establishes its asymptotic behavior under regular measures, and shows how it leads naturally to effective and implementable randomness tests.

The main theoretical result shows that for Martin–Löf random sequences under constructive exchangeable measures, empirical entropy provides the correct normalization scale for descriptive complexity. In this setting, normalized complexity converges to its natural benchmark, while deviations signal algorithmic structure beyond symbol imbalance. A complementary pathological example demonstrates that regularity of the underlying measure is essential: when strong dependencies are invisible at the level of single-symbol marginals, empirical entropy alone no longer captures the true combinatorial constraints.

The entropy-normalized viewpoint developed here is closely related to earlier work in which algorithmic complexity, Martin–Löf tests, universal priors, and Ockham-type principles were studied within a unified framework [3,5,6]. A recurring theme in this literature is the distinction between combinatorial entropy and intrinsic descriptive complexity; see, for example, Kramosil [7] and Calude [8], where information content is treated as a structural property not reducible to symbol frequencies alone.

The present approach complements these developments by making the combinatorial baseline explicit at the level of finite words. It thereby connects classical counting arguments, algorithmic randomness, and empirical entropy in a single framework. The normalization also aligns naturally with process-based and conditional notions of complexity. Earlier work in Vidakovic [9] introduced process-based conditional complexity and information measures defined through differences in complexities, emphasizing the role of conditioning objects and generating mechanisms. From a related perspective, Stojanović and Vidakovic [10] studied effective combinatorial measures of complexity for binary words, showing that complexity is tightly constrained by word weight and that shell-typical behavior dominates.

By explicitly factoring out the combinatorial contribution of empirical symbol frequencies, the entropy-normalized formulation isolates intrinsic descriptive structure and operates directly within the framework of Kolmogorov complexity and algorithmic randomness. Moreover, the same normalization principle extends naturally to conditional and mutual settings, where empirical joint or conditional entropies replace marginal symbol frequencies as the appropriate baseline. These extensions are developed later in the paper and provide a unified bridge between effective combinational measures, conditional complexity, and entropy rates arising in information theory. The normalization is operational at finite sample sizes. Because the empirical baseline is explicit, one can replace K(·) by computable description-length upper bounds and obtain calibrated statistics for detecting within-shell regularity in observed binary data.

This paper introduces an empirical-entropy normalization of Kolmogorov complexity for finite binary words and infinite sequences, designed to factor out the dominant combinatorial effects of symbol frequencies. By comparing description length to the size of the corresponding empirical type class, the proposed normalization yields a dimensionless measure of algorithmic structure that is stable across lengths and compositions. Under regular constructive measures, the normalized complexity is shown to converge to its natural benchmark for Martin–Löf random sequences, while a counterexample demonstrates the necessity of regularity assumptions. Replacing Kolmogorov complexity with computable upper bounds leads to effective statistics and implementable randomness tests for finite data.

The paper is organized as follows. Section 2 introduces preliminaries and background material. Section 3 develops the normalization principle and states the main theorem. Section 3.2 contains the proof of the limit result, and Section 3.3 explains the role of regularity. Section 4 and Section 5 discuss algorithmic and applied interpretations. Section 6 develops higher-order baselines. Section 7 gives conditional and mutual normalizations, and Section 8 concludes.

## 2. Preliminaries

This section fixes notation and recalls the algorithmic randomness concepts used throughout. We maintain a strict separation between notation for finite binary words and for prefixes of infinite sequences, and we adopt a consistent convention for empirical frequencies, entropy, and combinatorial baselines.

### 2.1. Sample Space, Cylinders, and Domination Relations

Let $\Omega ={\left\{0,1\right\}}^{N}$ denote the space of all infinite binary sequences. For ω∈Ω, its prefix of length *m* is written

(1) $$\begin{array}{c} \omega^{m}=\omega_{1}\omega_{2}\ldots \omega_{m}. \end{array}$$

For a finite binary word $x=x_{1}x_{2}\ldots x_{\ell (x)}$, the associated cylinder set is

(2) $$\begin{array}{c} \Gamma_{x}=\left\{\omega \in \Omega :\omega^{\ell (x)}=x\right\}. \end{array}$$

Cylinder sets generate the Borel σ-algebra on Ω [11,12].

We identify finite binary words with nonnegative integers via the standard bijection

(3) $$\begin{array}{c} x\mapsto 2^{\ell (x)}-1+\sum_{i=1}^{\ell (x)}x_{i}\;2^{\ell (x)-i}, \end{array}$$

and, when no ambiguity arises, use the same symbol to denote a word and its corresponding integer.

Throughout the paper we compare quantities only up to additive constants. For functions a(·) and b(·) defined on a common domain, we write

(4) $$\begin{array}{c} a⪯b \end{array}$$

if there exists a constant *C* such that a(u)≤b(u)+C for all relevant *u*. The reverse relation is denoted by a⪰b, and a≍b denotes mutual domination. This convention is standard in algorithmic information theory and avoids repeated low-order terms.

### 2.2. Kolmogorov Complexity, Martin–Löf Tests, and Randomness

Let P denote the class of all partial recursive functions acting on finite binary strings. For F∈P, the Kolmogorov complexity of a finite word *x* relative to *F* is

(5) $$\begin{array}{c} K_{F}\left(x\right)=\left\{\begin{array}{cc} \min\{\ell (p):F(p)=x\}, & \mathrm{if}\;\mathrm{such}\;p\;\mathrm{exists},\\ \infty , & \mathrm{otherwise}. \end{array}\right. \end{array}$$

There exists a universal partial recursive function $F_{0}\in P$ such that for all G∈P,

(6) $$\begin{array}{c} K\left(x\right)\equiv K_{F_{0}}\left(x\right)⪯K_{G}\left(x\right). \end{array}$$

We interpret K(x) as a description length in bits. Comprehensive treatments may be found in Li and Vitányi [12], Downey and Hirschfeldt [13], Nies [14].

Let μ be a probability measure on Ω. A function *V* defined on finite words is called a Martin–Löf test with respect to a constructive measure μ if there exists a recursive function g(m)↓0 such that, for every integer m≥1,

(7) $$\begin{array}{c} \mu \left(\left\{\omega \in \Omega :\underset{r\geq 1}{\sup}V\left(\omega^{r}\right)\geq m\right\}\right)\leq g\left(m\right). \end{array}$$

There exists a universal Martin–Löf test *U* satisfying U(x)⪯V(x) for all such tests [11].

A sequence ω∈Ω is Martin–Löf random with respect to μ if

(8) $$\begin{array}{c} \forall \text{Martin-Löf}\;\mathrm{tests}V,\;\underset{r\geq 1}{\sup}V\left(\omega^{r}\right)<\infty , \end{array}$$

or, equivalently,

(9) $$\begin{array}{c} \underset{r\geq 1}{\sup}U\left(\omega^{r}\right)<\infty . \end{array}$$

### 2.3. Empirical Distributions, Entropy, and Shells

For a finite binary word *x* of length ℓ(x), let w(x) denote its weight, that is, the number of ones in *x*. Define

(10) $$\begin{array}{c} p\left(x\right)=\frac{w(x)}{\ell (x)},\;\nu_{\ell (x)}=\left(p\left(x\right),1-p\left(x\right)\right). \end{array}$$

For an infinite sequence ω∈Ω, let $w(\omega^{m})$ be the number of ones in its prefix $\omega^{m}$ and define

(11) $$\begin{array}{c} p_{m}\left(\omega \right)=\frac{w(\omega^{m})}{m},\;\nu_{m}\left(\omega \right)=\left(p_{m}\left(\omega \right),1-p_{m}\left(\omega \right)\right). \end{array}$$

We use the binary entropy, measured in bits per symbol,

(12) $$\begin{array}{c} H\left(\nu \right)=-p\log_{2}p-\left(1-p\right)\log_{2}\left(1-p\right), \end{array}$$

with the convention 0log0=0. For fixed integers (m,k), the shell (type class)

(13) $$\begin{array}{c} S\left(m,k\right)=\{x\in {\left\{0,1\right\}}^{m}:w\left(x\right)=k\} \end{array}$$

has cardinality mk, and hence

(14) $$\begin{array}{c} \log_{2}\left|S\left(m,k\right)\right|=mH\left(k/m\right)+O\left(\log m\right). \end{array}$$

The quantity $\ell \left(x\right)H\left(\nu_{\ell (x)}\right)$ therefore represents the natural combinatorial baseline for describing a word with empirical distribution $\nu_{\ell (x)}$. This scale is the benchmark against which Kolmogorov complexity is compared throughout the paper.

**Figure 1:**
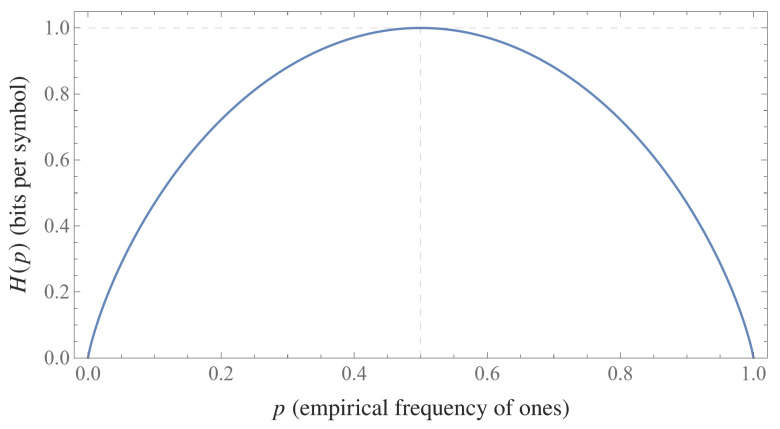
Binary empirical entropy $H\left(p\right)=-p\log_{2}p-\left(1-p\right)\log_{2}\left(1-p\right)$ as a function of the empirical frequency *p* of ones. The curve attains its maximum value of one bit at p=1/2 and decreases symmetrically as the symbol distribution becomes more imbalanced.

Figure 1 displays the binary entropy function H(p) and illustrates the degree of reduction caused by symbol imbalance.

## 3. The Adjusted Complexity *KA*(*x*)

This section formalizes the adjusted scale KA(x) and the ratio R(x), and makes precise the shell-based intuition motivating empirical entropy normalization. The key technical device is a shell counting statement (Step 3) converted into a Martin–Löf test (Step 4); the same logical pattern will be reused later when K(·) is replaced by computable description lengths and the resulting deficiency is turned into an implementable statistic (Section 6 and Section 8).

### 3.1. Definitions and the Normalization Principle

Throughout, w(x), p(x), $\nu_{\ell (x)}$, and $H(\nu_{\ell (x)})$ are as defined in Section 2, and $H(\nu_{\ell (x)})$ is measured in bits per symbol. The shell baseline log2|S(m,k)|=log2mk=mH(k/m)+O(logm) is also recalled in Section 2.

**Definition** **1.** *Define the entropy-normalized Kolmogorov complexity by*

(15) $$\begin{array}{c} KA\left(x\right)=\frac{K(x)}{H(\nu_{\ell (x)})}. \end{array}$$

*KA(x) is undefined when $H(\nu_{\ell (x)})=0$, that is, for constant words. For nonconstant words it is finite and provides a scale-free measure of descriptive complexity. In practice, constant words can be handled separately: they are maximally imbalanced and trivially compressible. The interesting regime is 0<p(x)<1, where empirical entropy provides a meaningful combinatorial baseline.*

Two standard baselines motivate (15). First, for fixed length ℓ(x)=n and fixed weight w(x)=k, the shell S(n,k) has cardinality nk, hence the shell-typical indexing cost satisfies

(16) log2nk=nH(k/n)+O(logn),

where the O(logn) term accounts for unavoidable self-delimiting overhead. Second, for any word *x* of length *n*, there exists a uniform coding scheme which, given *n*, first encodes the weight w(x) using O(logn) bits and then specifies *x* by indexing it within the shell S(n,w(x)). This yields the bound

K(x∣n)⪯log2nw(x)+logn.

Since K(x)=K(x∣n)+O(logn), the quantity log2nw(x) identifies the correct combinatorial baseline for the unconditional description length of *x*. Consequently, the empirical entropy $H(\nu_{\ell (x)})$ provides the appropriate per-symbol normalization, and the adjusted complexity rescales Kolmogorov complexity relative to the effective size of the corresponding empirical entropy shell.

**Definition** **2** (Normalized shell complexity)**.** *Let x be a finite binary word of length ℓ(x) with empirical distribution $\nu_{\ell (x)}$. Whenever $H(\nu_{\ell (x)})>0$, define the normalized shell complexity by*

(17) $$\begin{array}{c} R\left(x\right)=\frac{K(x)}{\ell \left(x\right)\;H\left(\nu_{\ell (x)}\right)}. \end{array}$$

*When $H(\nu_{\ell (x)})=0$ (constant words), we set R(x)=0 by convention and treat such words as trivially compressible edge cases.*

*The quantity R(x) measures the description length of x relative to the combinatorial baseline determined by its empirical distribution. For random objects generated under regular measures, R(x) is expected to be close to one. Values of R(x) substantially below one indicate compressibility arising from structural regularities beyond symbol imbalance. Later, in Section 5, Section 6 and Section 7, we return to R(x) and show how computable upper bounds on K(x) produce effective ratios that can be calibrated into within-shell tests.*

### 3.2. Regularity Assumptions and the Normalization Theorem

Let ω be Martin–Löf random with respect to a constructive exchangeable measure μ on $\Omega ={\left\{0,1\right\}}^{N}$. By de Finetti’s theorem [15], μ admits a mixture representation over Bernoulli product measures $\mu_{p}$, p∈[0,1], and for μ–typical sequences the empirical frequency of ones converges to a limiting parameter *p*. In the constructive setting, and as part of the regularity hypotheses underlying Theorem 1, we assume an effective form of this reduction: Martin–Löf randomness with respect to μ concentrates on a single Bernoulli component, so that ω is Martin–Löf random with respect to $\mu_{p}$ for some p∈[0,1]. This is the only point at which exchangeability enters the argument, and it explains why the natural normalization scale is the single-symbol empirical entropy $H(\nu_{m}\left(\omega \right))$.

**Theorem** **1.** *For such a sequence ω with limiting frequency p,*

(18) $$\begin{array}{c} \lim_{m\to \infty }\frac{KA(\omega^{m})}{m}=1,\;equivalently\;\lim_{m\to \infty }\frac{K(\omega^{m})}{m}=H\left(p\right). \end{array}$$

**Proof.**  The proof foregrounds a shell deficiency quantity, because the same deficiency reappears later as an effective test statistic once K(·) is replaced by its computable surrogates (Section 5). Write $k_{m}=w\left(\omega^{m}\right)$ and $p_{m}=k_{m}/m$. By definition $\nu_{m}\left(\omega \right)=\left(p_{m},1-p_{m}\right)$. Since ω is Martin–Löf random under the regular exchangeable μ, the empirical frequency converges,

(19) $$\begin{array}{c} p_{m}\to p, \end{array}$$

and therefore continuity of the binary entropy gives

(20) $$\begin{array}{c} H(\nu_{m}\left(\omega \right))\to H\left(p\right). \end{array}$$

Consequently,

(21) $$\begin{array}{c} \frac{KA(\omega^{m})}{m}=\frac{K(\omega^{m})}{m\;H(\nu_{m}\left(\omega \right))} \end{array}$$

converges to 1 if and only if

(22) $$\begin{array}{c} \frac{K(\omega^{m})}{m}\to H\left(p\right). \end{array}$$

We prove (22) by comparing $K(\omega^{m})$ to the shell baseline log2mkm and showing that the per-symbol difference vanishes. The argument has two parts: a constructive upper bound on $K(\omega^{m})$ via shell coding (Step 2), and a deficiency control obtained from a counting lemma and a Martin–Löf test construction (Steps 3 and 4). Theorem 1 should be read as an effective almost sure statement: ordinary almost sure convergence under μ is replaced by convergence along every Martin–Löf random sequence.

Step 0: Shells and the baseline.

For integers (m,k) define the shell

(23) $$\begin{array}{c} S\left(m,k\right)=\{x\in {\left\{0,1\right\}}^{m}:\;w\left(x\right)=k\}, \end{array}$$

so |S(m,k)|=mk. The quantity log2mk is the optimal indexing cost for words in S(m,k), up to additive O(logm) overhead.

Step 1: Stirling approximation yields the correct shell rate. Let $k=k_{m}$ and $p_{m}=k_{m}/m$. Standard Stirling bounds imply (16), where the remainder term O(logm) is explicit and uniform across the central range of $k_{m}$. Since $p_{m}\to p$, we obtain
  (24) 1mlog2mkm→H(p).

This is the target rate for $K(\omega^{m})/m$.

Step 2: An explicit upper bound on $K(\omega^{m})$ via shell coding. Fix *m* and $k=k_{m}$. There is a computable bijection between S(m,k) and {0,1,…,mk−1} given by lexicographic indexing. Hence there exists a prefix-free code that describes any x∈S(m,k) by (i) a self-delimiting description of (m,k) and (ii) the index of *x* within S(m,k). By arguments following Theorem 1, this yields a uniform bound
  (25) K(x)⪯log2mk+O(logm),
  for all words *x* of length *m* and weight *k*. Applying this to $x=\omega^{m}$, dividing by *m*, and using (24) yields
  (26) $$\begin{array}{c} \underset{m\to \infty }{lim sup}\frac{K(\omega^{m})}{m}\leq H\left(p\right). \end{array}$$

Step 3: Large deficiency inside a shell is rare. Define the shell deficiency of a word *x* of length *m* and weight *k* by
  (27) d(m,k;x)=log2mk−K(x).

Fix (m,k) and an integer t≥1, and consider the set

(28) $$\begin{array}{c} A_{m,k}\left(t\right)=\left\{x\in S\left(m,k\right):\;d\left(m,k;x\right)\geq t\right\}. \end{array}$$

Then

(29) |Am,k(t)|≤2−tmk.

Indeed, d(m,k;x)≥t is equivalent to K(x)≤log2mk−−t. There are at most 2log2mk−t programs of length at most log2mk−t and hence at most that many distinct outputs. Therefore at most a $2^{-t}$ fraction of the shell can have deficiency at least *t*.

Step 4: Turning (27) into a Martin-Löf test. For each m≥1 and each t≥1, define the set of sequences whose prefix of length *m* has deficiency at least *t* by
  (30) $$\begin{array}{c} B_{m}\left(t\right)=\left\{\omega \in \Omega :\;d\left(m,w\left(\omega^{m}\right);\omega^{m}\right)\geq t\right\}. \end{array}$$

We bound the probability of $B_{m}\left(t\right)$ under the Bernoulli measure $\mu_{p}$. For any word *x* of length *m* and weight *k*,

(31) $$\begin{array}{c} \mu_{p}\left(\Gamma_{x}\right)=p^{k}{\left(1-p\right)}^{m-k}. \end{array}$$

Summing over *k* and then over the atypical set $A_{m,k}\left(t\right)$ within each shell yields

(32) μp(Bm(t))=∑k=0m∑x∈Am,k(t)μp(Γx)=∑k=0m|Am,k(t)|pk(1−p)m−k≤∑k=0m2−tmkpk(1−p)m−k=2−t(p+(1−p))m=2−t.

Define a global test by weighting over *m* in a computable way, for example

(33) $$\begin{array}{c} U_{t}=\underset{m\geq 1}{⋃}B_{m}\;\left(t+2\log_{2}\left(m+1\right)\right). \end{array}$$

Using (32) with *t* replaced by $t+2\log_{2}\left(m+1\right)$ gives

(34) $$\begin{array}{ccc} \mu_{p}\left(U_{t}\right) & \leq & \sum_{m\geq 1}\mu_{p}\;\left(B_{m}\;\left(t+2\log_{2}\left(m+1\right)\right)\right)\leq \sum_{m\geq 1}2^{-(t+2\log_{2}\left(m+1\right))}\\ & = & 2^{-t}\sum_{m\geq 1}{\left(m+1\right)}^{-2}<c\;2^{-t}, \end{array}$$

for an absolute constant *c*. The family ${\left\{U_{t}\right\}}_{t\geq 1}$ is uniformly recursively enumerable (because K(x) is upper semicomputable, hence the predicate d(m,k;x)≥r is effectively enumerable) and therefore constitutes a Martin–Löf test with respect to $\mu_{p}$. Since ω is Martin–Löf random with respect to $\mu_{p}$, it avoids (i.e., passes) this test. Hence, there exists a constant $t_{0}$ such that for all sufficiently large *m*,

(35) $$\begin{array}{c} d\left(m,k_{m};\omega^{m}\right)<t_{0}+2\log_{2}\left(m+1\right). \end{array}$$

Equivalently,

(36) log2mkm−K(ωm)=O(logm).

Dividing by *m* gives

(37) 1mlog2mkm−K(ωm)→0.

Step 5: Conclusion. Combine (24) and (37):

(38) K(ωm)m=1mlog2mkm−1mlog2mkm−K(ωm)→H(p)−0=H(p).

Therefore, using (20),

(39) $$\begin{array}{c} \frac{KA(\omega^{m})}{m}=\frac{K(\omega^{m})}{m\;H(\nu_{m}\left(\omega \right))}\to 1, \end{array}$$

as claimed. □

**Remark** **1.** *The core of the argument is a deficiency control: within each shell, words with a large deficiency log2mk−K(x) form an exponentially small subset, and Martin–Löf randomness rules out infinitely many large deficiencies. This difference-of-complexities logic parallels the process-based information measures developed in [9,16], where information is defined via differences of conditional process complexities and is tied to randomness properties of sequences. In later sections we reuse this logic by replacing K(x) with computable description lengths, thereby turning the deficiency into a test statistic; see, in particular, the effective ratios in Section 5 and the consolidated testing rules in Section 7.*

### 3.3. Non-Ergodic and Pathological Measures

The regularity assumptions imposed on the underlying measure μ are essential for the validity and interpretability of Theorem 1. The key point is that the baseline used in the theorem is the single-symbol empirical entropy $H(\nu_{m}\left(\omega \right))$, which captures marginal symbol frequencies but ignores higher-order constraints. If the generating mechanism enforces strong dependencies while preserving an apparently balanced marginal distribution, then $H(\nu_{m}\left(\omega \right))$ can be close to 1 even though the true combinatorial growth rate of admissible strings is smaller. This is precisely the setting in which a higher-order normalization, based on empirical block entropies or conditional entropies, becomes necessary. This observation is echoed later when we introduce conditional empirical baselines in Section 6.

**Example** **1.** *To illustrate the point concretely, consider a process that emits blocks 00, 01, and 11 with equal probability and never produces the block 10. More precisely, define a measure μ on Ω supported on sequences ω such that for every k≥1 the length-2 block $\left(\omega_{2k-1},\omega_{2k}\right)$ belongs to {00,01,11}. This measure is highly constrained and is not exchangeable and not i.i.d.*

*For μ-typical sequences, the marginal frequency of ones is still 1/2, since each block has expected number of ones equal to 1, so the empirical distribution satisfies*

(40) $$\begin{array}{c} \nu_{m}\left(\omega \right)\to \left(1/2,1/2\right),\;H\left(\nu_{m}\left(\omega \right)\right)\to 1. \end{array}$$

*However, the number of admissible length-m words is of order $3^{m/2}$ (up to a factor depending on parity), since there are 3 choices for each disjoint pair. Thus the true combinatorial growth rate of the support (in bits/symbol) is*

(41) $$\begin{array}{c} \frac{1}{m}\log_{2}\left(3^{m/2}\right)=\frac{1}{2}\log_{2}3<1. \end{array}$$

*For μ-random sequences ω (random with respect to this constrained block measure), the Kolmogorov complexity of $\omega^{m}$ tracks the logarithm of the number of admissible prefixes, and therefore one expects*

(42) $$\begin{array}{c} \frac{K(\omega^{m})}{m}\to \frac{1}{2}\log_{2}3, \end{array}$$

*while at the same time $H(\nu_{m}\left(\omega \right))\to 1$. Consequently,*

(43) $$\begin{array}{c} \frac{K(\omega^{m})}{m\;H(\nu_{m}\left(\omega \right))}\to \frac{\left(1/2\right)\log_{2}3}{1}=\frac{1}{2}\log_{2}3, \end{array}$$

*which is strictly less than* 1*.*

This example shows that the limit theorem for entropy normalization may fail when the underlying measure has dependence structure that is invisible to the single-symbol empirical distribution. It also clarifies what the regularity assumptions accomplish: in the exchangeable i.i.d. mixture regime, the one-bit empirical entropy is the correct baseline, whereas in constrained or non-ergodic regimes it may be necessary to replace $H(\nu_{m})$ by block entropies, empirical conditional entropies, or other higher-order empirical summaries in order to obtain a stable and interpretable normalization. For this reason the conditional normalization developed later in Section 6 is not merely a formal extension; it is a practical response to precisely this type of hidden dependence.

## 4. Algorithmic Interpretation of Entropy Normalization

The normalization introduced in Section 3 has a natural interpretation from the standpoint of algorithmic information theory. The guiding idea is that Kolmogorov complexity should be assessed relative to the effective size of the empirical type class determined by observed symbol frequencies, rather than relative to word length alone. This section clarifies the meaning of the adjusted complexity KA(x) and the ratio R(x) and explains how they relate to randomness deficiency, Martin–Löf tests, and classical information-theoretic principles.

### 4.1. Complexity Relative to Empirical Type Classes

For a finite binary word *x* of length ℓ(x) and weight w(x), the shell S(ℓ(x),w(x)) represents the full collection of words sharing the same empirical distribution $\nu_{\ell (x)}$. As recalled in Section 2, the logarithm of the shell size,

(44) $$\begin{array}{c} \log_{2}\left|S\left(\ell \left(x\right),w\left(x\right)\right)\right|=\ell \left(x\right)\;H\left(\nu_{\ell (x)}\right)+O\left(\log\ell \left(x\right)\right), \end{array}$$

is the natural combinatorial baseline for describing such words.

From this perspective, raw Kolmogorov complexity K(x) conflates two distinct contributions. The first is the unavoidable combinatorial cost imposed by symbol frequencies, reflected in the size of the shell. The second is intrinsic algorithmic structure, corresponding to regularities that distinguish *x* from a typical element of its shell. The normalization embodied in KA(x) and R(x) removes the first contribution and isolates the second.

In particular, the ratio R(x), introduced in Definition 2, can be interpreted as a dimensionless measure of descriptive complexity relative to the empirical type class. Values of R(x) close to one indicate shell-typical behavior, while values substantially below one signal the presence of additional structure beyond symbol imbalance.

A closely related quantity is the shell deficiency

(45) d(ℓ(x),w(x);x)=log2ℓ(x)w(x)−K(x),

introduced in the proof of Theorem 1. This deficiency measures the extent to which *x* admits a shorter description than that of a typical element of its shell.

The counting argument in Step 3 of the proof shows that, for fixed (ℓ(x),w(x)), only an exponentially small fraction of words can exhibit deficiency exceeding a given threshold. When this observation is combined with the Martin–Löf test construction in Step 4, it implies that large deficiencies cannot occur infinitely often along prefixes of a Martin–Löf random sequence under a regular measure.

From this viewpoint, the convergence result in Theorem 1 states that, for random sequences, the shell deficiency grows at most logarithmically, while the shell baseline grows linearly. The adjusted complexity therefore captures the correct asymptotic scale of description length relative to empirical entropy.

### 4.2. Relation to Martin–Löf Randomness and Effective Tests

The normalization principle also clarifies the connection between Kolmogorov complexity and Martin–Löf randomness. Classical characterizations of randomness express the incompressibility of prefixes in terms of deviations of $K(\omega^{m})$ from *m*. The results of Section 3 show that, under regular exchangeable measures, the appropriate benchmark is not *m* but $mH(\nu_{m}\left(\omega \right))$.

Thus, randomness relative to a nonuniform Bernoulli source manifests itself as incompressibility relative to the empirical entropy scale. In this sense, empirical entropy plays the same role for nonuniform sources that word length plays for the fair coin.

This observation is essential for the development of effective tests in later sections. Once K(x) is replaced by computable upper bounds, the same deficiency-based logic yields implementable statistics that compare observed description lengths to empirical entropy baselines. These statistics inherit their interpretability from the algorithmic framework developed here.

Entropy-normalized complexity also aligns naturally with universal prior and minimum description length principles. In Bayesian and MDL settings, description length is often decomposed into a model cost and a data cost, with the latter reflecting combinatorial variability. The empirical entropy baseline plays an analogous role here, representing the cost of encoding data given its empirical composition, while the residual complexity reflects algorithmic structure.

Related ideas appear in earlier work on universal priors, Ockham-type principles, and process-based measures of information, where information is defined through differences of complexities rather than absolute magnitudes. From this perspective, KA(x) can be viewed as a normalized difference between description length and combinatorial entropy, providing a scale-free measure of intrinsic structure.

Finally, it is important to emphasize that the normalization developed here operates at the level of finite words. No asymptotic limit is required to define KA(x) or R(x), and the empirical entropy baseline is explicit and computable from the observed data. This finite-sample grounding is crucial for applications, where only finite strings are available and asymptotic approximations may be unreliable.

At the same time, the examples in Section 3.3 show that single-symbol entropy is not always sufficient to capture combinatorial constraints imposed by the generating mechanism. This motivates the conditional and block-based normalizations developed in Section 6 and Section 7, where empirical joint and conditional entropies replace marginal symbol frequencies as the appropriate baselines.

In summary, Section 3 establishes empirical entropy as the correct normalization scale for descriptive complexity under regular measures, while the present section interprets this normalization algorithmically and prepares the ground for effective surrogates and practical tests developed later in the paper.

## 5. Statistical, Applied, and Effective Uses of Adjusted Complexity

The entropy-normalized complexity framework admits both conceptual and practical interpretations once the abstract shell argument used in the proof of Theorem 1 is translated back to finite data. A central summary statistic is the normalized shell complexity R(x) from Definition 2, which compares the descriptive cost of a word to the combinatorial baseline implied by its empirical distribution. Values of R(x) close to one indicate that *x* is algorithmically typical relative to its empirical shell, while values substantially below one signal algorithmic structure beyond what symbol frequencies alone can explain. This normalization is essential in applications where the proportion of ones varies widely across samples, as it prevents sparsity or imbalance from being misinterpreted as regularity.

The abstract counting procedure used in Step 3 in the proof of Theorem 1 may already be read as a proto-statistical statement: within a fixed empirical shell, large deficiencies are exponentially rare. The present section explains how this principle becomes operational when combined with effective (computable) upper bounds on Kolmogorov complexity. Potential applications include randomness testing, complexity-based hypothesis testing, assessment of structure in symbolic sequences, analysis of biological and genomic data, and evaluation of binary patterns arising from thresholding procedures such as wavelet coefficient selection.

### 5.1. Shells, Typicality, and the Deficiency Viewpoint

Fix a word length *n* and a weight *k*. As recalled in Section 2 and used repeatedly in Section 3, the shell S(n,k) is the type class of all words of length *n* with weight *k*. The baseline descriptive cost for shell-typical words is log2nk, which is nH(k/n) up to the unavoidable O(logn) self-delimiting overhead.

Normalized shell typicality is captured by R(x), as introduced in Definition 2. Equivalently, the central object in the proof of Theorem 1 is the shell deficiency

d(n,k;x)=log2nk−K(x),

which measures how much shorter the description of *x* is than what the shell size alone would suggest.

In the uncomputable setting, large deficiency events are organized into a Martin–Löf test. In the effective setting, the same idea yields implementable statistics once K(·) is replaced by a computable surrogate.

### 5.2. Some Effective Surrogates for Kolmogorov Complexity

Kolmogorov complexity is not computable, so neither KA(x) nor R(x) can be evaluated exactly on observed data. The standard remedy is to work with a total recursive upper bound $K_{\mathrm{eff}}\left(x\right)$ satisfying

$$K\left(x\right)⪯K_{\mathrm{eff}}\left(x\right),$$

with an additive constant depending only on the chosen coding scheme. This turns the theoretical ratio R(x) into an effective test statistic that can be computed.

Three canonical choices illustrate the mechanism.

(i) Literal description length. The most basic computable bound is the literal code

$$K_{\mathrm{len}}\left(x\right)=\ell \left(x\right)=n,$$

which outputs *x* verbatim. This bound is deliberately crude but universally available.

(ii) Combinatorial (shell) description length. A stronger computable bound exploits the empirical shell structure. One first encodes n=ℓ(x) and the weight w(x) (both with O(logn) bits) and then indexes *x* within its shell. This yields

Kcomb(x)=log2nw(x)+O(logn).

This is the operational counterpart of the shell coding argument used in Step 2 of the proof of Theorem 1.

(iii) Lempel–Ziv (LZ76) complexity. A widely used effective surrogate is based on the incremental parsing scheme proposed in [17]. Given a finite binary word $x=x_{1}x_{2}\cdots x_{n}$, the LZ76 algorithm scans *x* from left to right and decomposes it into a sequence of distinct phrases, where each new phrase is defined as the shortest substring that has not appeared previously as a contiguous block in the already parsed prefix. Let $c_{\mathrm{LZ}}\left(x\right)$ denote the total number of phrases produced by this parsing.

The associated Lempel–Ziv complexity is defined as

$$K_{\mathrm{LZ}}\left(x\right)=c_{\mathrm{LZ}}\left(x\right)\;\log_{2}n,$$

up to lower-order additive terms, and is computable in linear time.

Operationally, $K_{\mathrm{LZ}}\left(x\right)$ measures the rate at which new patterns appear in the sequence. Highly regular sequences generate only a small number of phrases, whereas algorithmically complex or random-looking sequences produce $c_{\mathrm{LZ}}\left(x\right)$ of order n/logn. Moreover, for stationary ergodic sources, the normalized quantity

$$\frac{c_{\mathrm{LZ}}\left(x\right)\log_{2}n}{n}$$

converges almost surely to the entropy rate, which further justifies the use of Lempel–Ziv complexity as an effective surrogate for Kolmogorov complexity.

Other effective surrogates for Kolmogorov complexity include compression-based description lengths derived from practical lossless compressors such as gzip, bzip2, and PPM, MDL/universal coding, as well as entropy-based plug-in and block-entropy estimators, all of which provide computable upper bounds or approximations to K(x) and are widely used in practice [12,18,19,20,21,22].

### 5.3. Effective Normalized Ratios and Decision Thresholds

Given any chosen computable bound $K_{\mathrm{eff}}\left(x\right)$, define the effective normalized ratio

$$R_{\mathrm{eff}}\left(x\right)=\frac{K_{\mathrm{eff}}\left(x\right)}{nH(\nu_{n})},\;n=\ell \left(x\right),$$

whenever $H(\nu_{n})>0$.

The denominator represents the logarithmic size of the empirical Bernoulli shell, while the numerator is a concrete description length. Thus, $R_{\mathrm{eff}}\left(x\right)$ compares the observed descriptive cost of *x* to the cost predicted by its empirical entropy.

The associated effective entropy deficiency is

$$d_{A}^{\mathrm{eff}}\left(x\right)=nH\left(\nu_{n}\right)-K_{\mathrm{eff}}\left(x\right).$$

A word is flagged as structurally non-typical relative to its empirical shell when the effective deficiency exceeds a user-chosen threshold m≥1, that is, when

$$d_{A}^{\mathrm{eff}}\left(x\right)\geq m.$$

This criterion can be expressed directly in terms of the ratio:

(46) $$\begin{array}{ccc} d_{A}^{\mathrm{eff}}\left(x\right)\geq m & ⟺ & K_{\mathrm{eff}}\left(x\right)\leq nH\left(\nu_{n}\right)-m⟺R_{\mathrm{eff}}\left(x\right)\leq 1-\frac{m}{nH(\nu_{n})}. \end{array}$$

**Figure 2:**
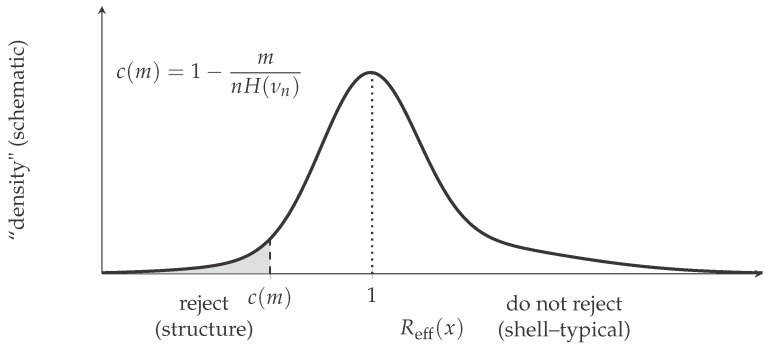
Schematic effective test based on the entropy-normalized ratio $R_{\mathrm{eff}}\left(x\right)=K_{\mathrm{eff}}\left(x\right)/\left(nH\left(\nu_{n}\right)\right)$. The gray region under the left tail corresponds to rejection of shell-typicality (structural regularity), with cutoff $c\left(m\right)=1-m/(nH\left(\nu_{n}\right))$; values near 1 are consistent with shell-typical behavior. The graph of “density” is schematic image of empirical distribution of $R_{\mathrm{eff}}\left(x\right)$ within the shell.

Figure 2 depicts this rule graphically: for fixed *m*, shell-typicality is rejected whenever

$$R_{\mathrm{eff}}\left(x\right)\leq c\left(m\right)=1-\frac{m}{nH(\nu_{n})}.$$

### 5.4. Statistical Interpretation and Finite Example

The logic behind the threshold is the same as in Step 3 in the proof of Theorem 1, except that K(·) is replaced by a computable upper bound. Within a fixed empirical shell, only an exponentially small fraction of words can have deficiency at least *m*. The parameter *m* therefore plays the role of a significance level that is intrinsically calibrated on the entropy scale and is not confounded by symbol imbalance. This is precisely what Figure 2 is intended to convey.

**Example** **2** (finite calculation under crude effective bounds)**.** *Consider the binary word*

(47) $$\begin{array}{c} x=01010001001000001010000100000100001, \end{array}$$

*of length n=35 and weight w(x)=9. Its empirical entropy is*

(48) $$\begin{array}{c} H\left(\nu_{n}\right)\approx 0.82,\;nH\left(\nu_{n}\right)\approx 28.78\;bits. \end{array}$$

*Using the literal bound yields $R_{\mathrm{len}}\left(x\right)\approx 1.22$, $R_{\mathrm{comb}}\left(x\right)\approx 1.09$, while using the Lempel–Ziv complexity measure yields $R_{\mathrm{LZ}76}\left(x\right)\approx 1.60$. If we choose m=5, then the rejection threshold in *(46) *is c(5)≈0.83, so randomness is not rejected under this crude effective coding schemes.*

*The interpretation is the intended one: entropy normalization ensures that the test compares x relative to the correct combinatorial baseline and does not falsely label a word as “structured” merely because it is sparse or imbalanced. More refined effective bounds $K_{\mathrm{eff}}$ (for example, bounds induced by modern lossless compressors) will typically move $R_{\mathrm{eff}}\left(x\right)$ closer to the ideal ratio R(x), and consequently increase sensitivity to genuine algorithmic structure while maintaining the within-shell calibration provided by $nH(\nu_{n})$.*

**Example** **3** (modified Thue–Morse under an LZ76 effective bound)**.** *Consider the modified Thue–Morse word*

(49) $$\begin{array}{ccc} x & = & 001010001000001010000010001010001000001000101000001\\ & & \ldots 0100010000010100000100010100000101000100000100010, \end{array}$$

*obtained by taking a segment of the Thue–Morse sequence [23] of length 100, and then setting every symbol equal to* 1 *in an even position to* 0*. For this word,*

$$\begin{array}{c} \ell \left(x\right)=n=100,\;w\left(x\right)=25,\;p\left(x\right)=0.25,\;\nu_{n}=\left(0.25,0.75\right). \end{array}$$

*The empirical (binary) Shannon entropy is*

(50) $$\begin{array}{c} H\left(\nu_{n}\right)\approx 0.81,\;nH\left(\nu_{n}\right)\approx 81.13\;bits. \end{array}$$

*For the word in *(49)*, the LZ76 factor count is*

(51) $$\begin{array}{c} c_{\mathrm{LZ}}\left(x\right)=10,\;K_{\mathrm{LZ}}\left(x\right)=10\log_{2}\left(100\right)\approx 66.44\;bits. \end{array}$$

*Hence, the effective entropy-normalized ratio is*

(52) $$\begin{array}{c} R_{\mathrm{eff}}\left(x\right)=\frac{K_{\mathrm{LZ}}\left(x\right)}{nH(\nu_{n})}\approx \frac{66.44}{81.13}\approx 0.82. \end{array}$$

*With m=5, the rejection threshold is*

(53) $$\begin{array}{c} c\left(5\right)=1-\frac{5}{nH(\nu_{n})}\approx 1-\frac{5}{81.13}\approx 0.94. \end{array}$$

*Since $R_{\mathrm{eff}}\left(x\right)\approx 0.82<c\left(5\right)\approx 0.94$, the LZ76 effective test rejects shell-typicality at level m=5 for this modified Thue–Morse word. The point is that, although the empirical entropy baseline correctly accounts for the imbalance p(x)=0.25, the LZ76 description length still falls substantially below $nH(\nu_{n})$, reflecting algorithmic regularities (here inherited from the Thue–Morse construction) that go beyond single-symbol frequencies.*

The pathological construction in Section 3.3 shows that single-symbol empirical entropy can fail when dependence constraints are invisible at the marginal level. The natural remedy is to enlarge the empirical baseline by moving to conditional or joint empirical entropies. The next section does exactly this, replacing $H(\nu_{n})$ by conditional and joint empirical quantities so that side information and dependence structure can be incorporated systematically.

## 6. Conditional and Higher-Order Entropy Normalization

Section 3, Section 4 and Section 5 establish empirical single-symbol entropy as the correct normalization scale for descriptive complexity under regular exchangeable measures, and show how this normalization leads naturally to effective statistics and implementable tests for finite data. The pathological example in Section 3.3, however, demonstrates that single-symbol empirical entropy may fail to capture the true combinatorial constraints imposed by the generating mechanism when strong dependencies are present.

The purpose of this section is to extend the normalization principle beyond marginal symbol frequencies. We show that the same shell-based and deficiency-based logic applies when empirical conditional or joint entropies are used as the baseline. This extension is not merely formal: it is required whenever dependence structure is invisible at the level of single-symbol marginals but becomes apparent once conditioning information is taken into account.

### 6.1. Motivation: When Marginal Entropy Is Insufficient

Recall the block-constrained process introduced in Section 3.3, where admissible length–2 blocks are {00,01,11} and the block 10 never appears. For sequences generated by this process, the empirical marginal distribution satisfies

$$\nu_{m}\left(\omega \right)\to \left(1/2,1/2\right),\;H\left(\nu_{m}\left(\omega \right)\right)\to 1,$$

even though the true combinatorial growth rate of admissible strings is strictly smaller, namely $\left(1/2\right)\log_{2}3$ per symbol. As shown there, this leads to a limiting normalized ratio strictly less than one.

This phenomenon illustrates a general point. Empirical marginal entropy captures only first-order frequency information. When the generating mechanism enforces higher-order constraints, the effective size of the admissible type class may be substantially smaller than what marginal entropy suggests. In such cases, the appropriate normalization must reflect conditional or joint structure rather than marginal frequencies alone.

### 6.2. Conditional Empirical Entropy as a Combinatorial Baseline

The empirical conditional entropy of order *r* measures how unpredictable a symbol of the word *x* is when one is allowed to use the preceding *r* symbols as context.

Let $x=x_{1}x_{2}\cdots x_{n}$ be a finite binary word. Fix an integer order r≥1 and consider empirical blocks of length r+1. For each binary string *u* of length *r*, define

$$N\left(u\right)=\#\left\{i:\;r+1\leq i\leq n,\;x_{i-r}^{i-1}=u\right\},\;\;\;\;N\left(u,b\right)=\#\left\{i:\;r+1\leq i\leq n,\;x_{i-r}^{i-1}=u,\;x_{i}=b\right\},$$

so that $\sum_{u\in {\left\{0,1\right\}}^{r}}N\left(u\right)=n-r$. The empirical conditional distribution of $x_{i}$ given the preceding block *u* is

$$\hat{p}\left(b\;|\;u\right)=\frac{N(u,b)}{N(u)}.$$

The empirical conditional entropy of order *r* is defined by

(54) $$\begin{array}{ccc} H_{r}\left(x\right) & = & -\sum_{u\in {\left\{0,1\right\}}^{r}}\frac{N(u)}{n-r}\sum_{b\in \{0,1\}}\hat{p}\left(b\;|\;u\right)\log_{2}\hat{p}\left(b\;|\;u\right), \end{array}$$

with the convention 0log0=0. This quantity measures the average uncertainty of a symbol among the n−r positions for which an (r+1)-block is defined.

Some authors divide by *n* rather than n−r, which produces the scaled value $\frac{n-r}{n}H_{r}\left(x\right)$. For fixed *r* this distinction is asymptotically negligible, but at short lengths it is preferable to average over the positions actually contributing to the empirical transition counts.

From a combinatorial standpoint, $\left(n-r\right)H_{r}\left(x\right)$ represents the logarithmic size (to first order) of the empirical conditional type class: the collection of words that share the same empirical conditional transition counts up to order *r*. Just as $nH(\nu_{n})$ arises from counting shells based on symbol frequencies, $\left(n-r\right)H_{r}\left(x\right)$ arises from counting admissible words subject to fixed empirical transition statistics.

**Example** **4.** *For the binary word x in (47) from Example 2, the empirical conditional entropies $H_{r}\left(x\right)$ for orders r=0,1,2,3 are*

$$H_{0}\left(x\right)\approx 0.82240,\;H_{1}\left(x\right)\approx 0.71162,\;H_{2}\left(x\right)\approx 0.67920,\;and\;H_{3}\left(x\right)\approx 0.66640.$$

### 6.3. Conditional Normalization of Kolmogorov Complexity

The conditional normalization principle mirrors the marginal case. Define the conditional entropy-normalized complexity of order *r* by

(55) $$\begin{array}{c} KA_{r}\left(x\right)=\frac{K(x)}{H_{r}\left(x\right)}, \end{array}$$

whenever $H_{r}\left(x\right)>0$. Similarly, define the normalized conditional ratio

(56) $$\begin{array}{c} R_{r}\left(x\right)=\frac{K(x)}{\left(n-r\right)H_{r}\left(x\right)}. \end{array}$$

The interpretation is analogous to that of KA(x) and R(x). The denominator represents the effective combinatorial baseline implied by empirical conditional structure, while the numerator reflects descriptive complexity. Values of $R_{r}\left(x\right)$ close to one indicate typicality relative to the empirical conditional type class, while substantially smaller values signal algorithmic structure beyond what the observed conditional frequencies alone can explain.

Under regularity assumptions analogous to those in Section 3, conditional normalization recovers the correct entropy rate. For stationary ergodic sources with finite memory, empirical conditional entropies converge almost surely to the true entropy rate. In such settings one expects

$$\frac{K(\omega^{m})}{mH_{r}\left(\omega^{m}\right)}\to 1$$

for Martin–Löf random sequences, provided *r* is chosen large enough to capture the dependence structure of the source.

### 6.4. Effective Conditional Statistics

As in Section 5, Kolmogorov complexity can be replaced by computable upper bounds to obtain effective conditional statistics. Given a computable description length $K_{\mathrm{eff}}\left(x\right)$, define

$$R_{r,\mathrm{eff}}\left(x\right)=\frac{K_{\mathrm{eff}}\left(x\right)}{\left(n-r\right)H_{r}\left(x\right)},\;d_{r}^{\mathrm{eff}}\left(x\right)=\left(n-r\right)H_{r}\left(x\right)-K_{\mathrm{eff}}\left(x\right).$$

Within a fixed empirical conditional type class, large deficiencies are exponentially rare, and thresholds on $d_{r}^{\mathrm{eff}}\left(x\right)$ yield calibrated tests of typicality relative to the empirical conditional structure. This provides a principled way to incorporate memory effects or contextual constraints into effective randomness testing.

The extensions developed in this section show that entropy normalization is not tied to single-symbol frequencies. Rather, it is a general principle: descriptive complexity should be assessed relative to the effective combinatorial size of the empirical type class determined by the available information. The appropriate entropy baseline depends on what aspects of structure are deemed relevant or observable.

## 7. Conditional and Mutual Adjusted Complexity

The entropy-normalized framework extends naturally to conditional and mutual settings, providing a principled way to factor out empirical joint structure in the presence of side information. As in the unconditional case, normalization separates purely combinatorial effects from algorithmic dependence.

Let $x,y\in {\left\{0,1\right\}}^{n}$ be finite binary words of common length *n*. Define the empirical joint frequencies by

$$\begin{array}{c} p_{ab}=\frac{1}{n}\#\left\{i:\;x_{i}=a,\;y_{i}=b\right\},\;a,b\in \left\{0,1\right\}. \end{array}$$

Let $p_{\cdot b}=\sum_{a}p_{ab}$ and $p_{a|b}=p_{ab}/p_{\cdot b}$ whenever $p_{\cdot b}>0$. The empirical conditional entropy is then

(57) $$\begin{array}{ccc} H_{\mathrm{emp}}\left(X|Y\right) & = & \sum_{b\in \{0,1\}}p_{\cdot b}\left(-\sum_{a\in \{0,1\}}p_{a|b}\log_{2}p_{a|b}\right). \end{array}$$

We define the conditional adjusted complexity and its normalized ratio by

(58) $$\begin{array}{ccc} KA(x|y) & = & \frac{K(x|y)}{H_{\mathrm{emp}}\left(X|Y\right)},\;R\left(x|y\right)=\frac{K(x|y)}{nH_{\mathrm{emp}}\left(X|Y\right)}, \end{array}$$

whenever $H_{\mathrm{emp}}\left(X|Y\right)>0$. The associated conditional entropy deficiency

(59) $$\begin{array}{ccc} d_{A}\left(x|y\right) & = & nH_{\mathrm{emp}}\left(X|Y\right)-K\left(x|y\right) \end{array}$$

is a special case of Levin’s randomness deficiency [4].

Here and throughout, the symbols *X* and *Y* do not denote abstract random variables generated by an underlying stochastic process. Instead, they refer to the empirical random variables induced by the fixed finite words $x=(x_{1},\ldots ,x_{n})$ and $y=(y_{1},\ldots ,y_{n})$: an index *i* is drawn uniformly from {1,…,n} and $X\left(i\right)=x_{i}$, $Y\left(i\right)=y_{i}$. All entropies are therefore computed with respect to the empirical joint distribution determined by (x,y). This convention is standard in the theory of types and empirical distributions [18] and is used explicitly when relating complexity to entropy rates [12].

Dependence between finite objects can also be studied through algorithmic mutual information. The algorithmic mutual information between two finite words *x* and *y* is defined, up to an additive constant, by

(60) $$\begin{array}{ccc} I(x:y) & ≍ & K(x)+K(y)-K(x,y), \end{array}$$

and measures the amount of algorithmic information shared by *x* and *y* [2,4,24]. From an empirical perspective, dependence is reflected in joint symbol frequencies. Letting *X* and *Y* denote the empirical random variables induced by $\left(x_{i},y_{i}\right)$, the empirical mutual entropy is

(61) $$\begin{array}{ccc} I_{\mathrm{emp}}\left(X;Y\right) & = & H_{\mathrm{emp}}\left(X\right)+H_{\mathrm{emp}}\left(Y\right)-H_{\mathrm{emp}}\left(X,Y\right). \end{array}$$

We define the mutual adjusted complexity by

(62) $$\begin{array}{c} KA\left(x:y\right)=\frac{I(x:y)}{I_{\mathrm{emp}}\left(X;Y\right)}, \end{array}$$

whenever $I_{\mathrm{emp}}\left(X;Y\right)>0$. This normalization factors out the purely statistical component of dependence and isolates algorithmic dependence beyond empirical correlation.

**Example** **5.** *We illustrate conditional normalization on a finite example, parallel to the unconditional calculation in Example 2. Let the binary word x be as in *(47)*. Relative to its unconditional shell, x does not exhibit substantial regularity beyond symbol imbalance.*

*Introduce periodic side information by letting*

$$\begin{array}{c} y_{0}=00100011, \end{array}$$

*and extending it periodically, truncating to length n=ℓ(x)=35 to obtain a word y. Forming the coordinatewise pairs $\left(x_{i},y_{i}\right)$ yields the joint counts in Table 1.*

*From Table 1, the empirical conditional probabilities are*

$$\begin{array}{c} P\left(X=1|Y=0\right)=\frac{6}{25},\;P\left(X=1|Y=1\right)=\frac{3}{10}, \end{array}$$

*with corresponding conditional entropies*

$$\begin{array}{c} H(X|Y=0)\approx 0.79,\;H(X|Y=1)\approx 0.88. \end{array}$$

*Here, (X,Y) are empirival variables induced by the pairs (x,y). Weighting by P(Y=0)=25/35 and P(Y=1)=10/35 gives*

$$\begin{array}{c} H_{\mathrm{emp}}\left(X|Y\right)=\frac{25}{35}\;H\left(X|Y=0\right)+\frac{10}{35}\;H\left(X|Y=1\right)\approx 0.82. \end{array}$$

*Thus, the conditional entropy baseline satisfies*

(63) $$\begin{array}{c} nH_{\mathrm{emp}}\left(X|Y\right)\approx 28.69\;bits,\;nH\left(\nu_{n}\right)\approx 28.78\;bits. \end{array}$$

*In this example, conditioning on the periodic word y reduces the entropy baseline only marginally (about 0.1 bits). Equivalently, the conditional shell determined by the joint counts has essentially the same logarithmic size as the unconditional shell. The conditioning partitions coordinates into the classes $\left\{i:\;Y_{i}=0\right\}$ and $\left\{i:\;Y_{i}=1\right\}$, and within each class the admissible patterns of x are governed by the corresponding empirical conditional type. Accordingly, $nH_{\mathrm{emp}}\left(X|Y\right)$ quantifies the logarithmic size of the conditional shell compatible with the observed joint structure.*

A natural effective conditional description length is obtained by coding *x* within this conditional shell, for example by separately indexing the locations of ones among the Y=0 coordinates and the Y=1 coordinates. The leading term of such a conditional shell index code is

log2256+log2103,

with an additional O(logn) overhead to describe the relevant counts and delimit the two subcodes. For the present short word (n=35), this lower-order overhead can be visible numerically; asymptotically it is negligible. In either case, the resulting effective ratio

$$R_{\mathrm{eff}}\left(x|y\right)=\frac{K_{\mathrm{eff}}\left(x|y\right)}{nH_{\mathrm{emp}}\left(X|Y\right)}$$

remains close to one, so the qualitative conclusion mirrors the unconditional case: relative to the conditional combinatorial baseline induced by *y*, the word *x* exhibits no substantial additional regularity beyond what is already explained by its empirical counts.

If *x* were nearly determined by *y*, then $H_{\mathrm{emp}}\left(X|Y\right)$ would be close to zero and a conditional shell code would achieve a much shorter description length, driving $R_{\mathrm{eff}}\left(x|y\right)$ far below one. Conditional entropy normalization therefore provides a within-shell comparison that separates genuine algorithmic structure in *x* from statistical constraints imposed by side information.

**Remark** **2** (Symmetry as a structural influence)**.** *Structural regularities may affect descriptive complexity beyond what is captured by symbol frequencies. For example, the binary word*

x=0000110000

*exhibits mirror symmetry, and thus admits a shorter description than a generic word with the same empirical entropy. In this sense, symmetry acts as an organizing constraint that reduces effective complexity without changing marginal frequencies. Related links between symmetry and entropy reduction are emphasized in [25,26].*

## 8. Discussion and Conclusions

The entropy-normalized complexity framework developed in this paper is guided by a simple organizing principle: descriptive structure should be assessed relative to the appropriate empirical combinatorial baseline. Kolmogorov complexity measures intrinsic algorithmic description length, while empirical entropy quantifies the size of the combinatorial class compatible with observed symbol frequencies. Normalizing complexity by empirical entropy separates these two effects and clarifies how much structure remains once trivial imbalance has been factored out.

This viewpoint provides a finite, local counterpart to the classical links between Shannon entropy, universal priors, and algorithmic complexity [27,28,29]. In particular, it makes explicit that entropy alone cannot capture algorithmic structure [7]: two words with identical empirical entropy may differ radically in descriptive complexity, and conversely, raw complexity values are not directly comparable across different empirical distributions. The adjusted quantities KA(x) and R(x) resolve this mismatch by placing description length on the correct empirical scale.

Several extensions and refinements arise naturally from this perspective. While the present development focuses on binary words and single-symbol empirical entropy, the normalization principle extends immediately to larger alphabets. More substantively, one may replace $H(\nu_{n})$ by block entropies, empirical conditional entropies, or other summaries that capture higher-order dependence. Such refinements become essential when symbol-level marginals do not reflect the true combinatorial constraints of the generating mechanism. The pathological example in Section 3.3 illustrates this vividly: when strong dependencies are invisible at the marginal level, a higher-order empirical baseline is required. The conditional normalization developed in Section 7 represents a first systematic step in this direction.

The framework also aligns naturally with ideas from algorithmic statistics. In this interpretation, the empirical distribution plays the role of a coarse statistical model, while the associated deficiency measures quantify residual algorithmic irregularity relative to that model. A more refined treatment could embed empirical, block, or conditional summaries into an explicit model class and interpret entropy-normalized deficiencies as stochasticity indices. This would connect the present framework more directly to minimal sufficient statistics and classical notions of randomness deficiency. The conditional and mutual constructions introduced here already point toward such a synthesis.

Computational considerations raise further questions. While Section 5 demonstrates how computable description lengths lead to implementable tests with controlled false-positive rates, systematic finite-sample calibration and power analysis under simple null models remain open problems. Addressing these issues would strengthen the bridge between algorithmic randomness theory and practical statistical methodology.

From an applied perspective, entropy normalization is particularly natural in settings where imbalance or sparsity is intrinsic rather than exceptional. In multiresolution representations, such as wavelet skeletons, thresholded coefficient patterns, or sparse symbolic encodings, imbalance is the default. Raw complexity measures tend to conflate sparsity with structure, whereas entropy-normalized complexity provides a principled way to compare descriptive content across scales. Extending the present framework to multiscale or hierarchical settings may therefore yield useful tools for analyzing structured signals and symbolic data.

Finally, a quantum analogue suggests itself. In Schumacher’s theory of quantum data compression, von Neumann entropy governs the compressibility of quantum ensembles [30]. A quantum-adjusted complexity would require replacing classical empirical entropy by state entropy and measuring algorithmic structure relative to density operators rather than symbol frequencies. Such a notion could separate structural quantum correlations from stochastic noise in mixed or entangled states, potentially strengthening conceptual links between algorithmic information theory, quantum coding, and quantum statistical inference.

In summary, entropy-normalized Kolmogorov complexity provides a flexible and unifying framework linking algorithmic information, empirical entropy, and statistical modeling. By explicitly factoring out the combinatorial contribution of empirical distributions, it yields quantities that are both theoretically interpretable and operationally meaningful. The resulting perspective clarifies the role of complexity in randomness, dependence, and structure detection, and opens several avenues for further theoretical development and applied use.

## Figures and Tables

**Table 1:** Joint counts for (x,y).

x∖y	0	1	Total
**0**	19	7	26
**1**	6	3	9
**Total**	25	10	35

## Data Availability

The original contributions presented in this study are included in the article. Further inquiries can be directed to the author.
